# Predicting brain activation maps for arbitrary tasks with cognitive encoding models

**DOI:** 10.1016/j.neuroimage.2022.119610

**Published:** 2022-09-03

**Authors:** Jonathon Walters, Maedbh King, Patrick G. Bissett, Richard B. Ivry, Jörn Diedrichsen, Russell A. Poldrack

**Affiliations:** a Department of Psychology, Stanford University, Stanford, CA, USA; b Department of Psychology, University of California Berkeley, Berkeley, CA, USA; c Helen Wills Neuroscience Institute, University of California Berkeley, Berkeley, CA, USA; d Brain and Mind Institute, Western University, London, Ontario, Canada; e Department of Computer Science, Western University, London, Ontario, Canada

**Keywords:** Encoding models, Computational modeling, Cognition, Ontologies, functional MRI

## Abstract

A deep understanding of the neural architecture of mental function should enable the accurate prediction of a specific pattern of brain activity for any psychological task, based only on the cognitive functions known to be engaged by that task. Encoding models (EMs), which predict neural responses from known features (e.g., stimulus properties), have succeeded in circumscribed domains (e.g., visual neuroscience), but implementing domain-general EMs that predict brain-wide activity for arbitrary tasks has been limited mainly by availability of datasets that 1) sufficiently span a large space of psychological functions, and 2) are sufficiently annotated with such functions to allow robust EM specification. We examine the use of EMs based on a formal specification of psychological function, to predict cortical activation patterns across a broad range of tasks. We utilized the Multi-Domain Task Battery, a dataset in which 24 subjects completed 32 ten-minute fMRI scans, switching tasks every 35 s and engaging in 44 total conditions of diverse psychological manipulations. Conditions were annotated by a group of experts using the Cognitive Atlas ontology to identify putatively engaged functions, and region-wise cognitive EMs (CEMs) were fit, for individual subjects, on neocortical responses. We found that CEMs predicted cortical activation maps of held-out tasks with high accuracy, outperforming a permutation-based null model while approaching the noise ceiling of the data, without being driven solely by either cognitive or perceptual-motor features. Hierarchical clustering on the similarity structure of CEM generalization errors revealed relationships amongst psychological functions. Spatial distributions of feature importances systematically overlapped with large-scale resting-state functional networks (RSNs), supporting the hypothesis of functional specialization within RSNs while grounding their function in an interpretable data-driven manner. Our implementation and validation of CEMs provides a proof of principle for the utility of formal ontologies in cognitive neuroscience and motivates the use of CEMs in the further testing of cognitive theories.

## 1. Introduction

A deep understanding of the neural architecture of mental function should enable the accurate prediction of a specific pattern of brain activity for any given psychological task, based only on the cognitive functions known to be engaged by that task. This type of prediction has been achieved in a number of specific domains using voxelwise encoding models (EMs), which aim to predict neural responses from a set of known features (such as stimulus features or computational model components) (Naselaris et al. 2011 ; Serences and Saproo 2012). These models have been particularly effective in visual neuroscience, where they have been used to characterize neuronal mechanisms of attention (e.g., Ester et al. 2016), motion perception (e.g., Vintch and Gardner, 2014), and natural image processing (e.g., Kay et al. 2008), among many others. They have also been applied in the context of language, where they have been used with models that embed linguistic stimuli in a low-dimensional space to predict patterns of brain activity for untrained words (Mitchell et al., 2008) and to create a cortical atlas of semantic space (Huth et al., 2016).

In the present work we examine the use of encoding models based on a formal specification of cognitive function, known as a cognitive ontology (Poldrack and Yarkoni 2016), to predict cortical activation patterns for cognitive tasks. In particular, we utilize the Cognitive Atlas (Poldrack et al., 2011), a knowledge base that defines a set of cognitive functions and the way that they are measured in various tasks. The resulting cognitive encoding models (CEMs) map from an annotation of the functions engaged by any particular task condition to activation at each location in the brain. The implementation and validation of such models provides a proof of principle for the utility of formal ontologies in cognitive neuroscience and motivates the use of this approach in the further testing of cognitive theories, which often vary in their predictions regarding the cognitive functions engaged by any given task.

The implementation of cognitive encoding models has been limited in large part by the availability of datasets that 1) are sufficiently broad to span a large space of cognitive functions, and 2) are sufficiently annotated with respect to these functions to allow robust specification of the encoding model. To address this challenge, we utilized the Multi-Domain Task Battery (King et al., 2019), in which 24 subjects completed 32 ~10-minute fMRI scans, switching tasks every 35 s and engaging in 44 total task conditions spanning a wide range of cognitive functions. Each of the task conditions was annotated by a set of experts using the Cognitive Atlas to identify the putatively engaged cognitive functions. We then fit CEMs based on task responses estimated from a set of regions spanning the entire neocortex (Schaefer et al., 2018), and assessed the degree to which these models could accurately predict brain responses on held-out task conditions.

Our work extends several recent studies that have attempted to decode cognitive functions using cognitive ontologies or to predict activation for novel tasks using cognitive relations between tasks. One recent study (Varoquaux et al., 2018) demonstrated the utility of a cognitive ontology for decoding concepts from patterns of brain activation, using population-level data aggregated across 30 fMRI studies (196 total experimental conditions) (Varoquaux et al., 2018). Training on many diverse conditions and accurately decoding concepts for unseen conditions provided strong evidence that ontology-based approaches may be useful for establishing selective associations between brain regions/networks and particular cognitive functions. However, the features in this study were relatively task-focused (e.g. stimulus or response features), unlike the function-focused features used in the present paper. Another recent study (Nakai and Nishimoto, 2020) showed the effectiveness of metadata-based features in an encoding model to predict brain activity and to decode tasks (instead of concepts), using subject-level data (103 total tasks) (Nakai and Nishimoto, 2020). This study also demonstrated the ability to predict activation patterns for unseen tasks, using a latent feature space derived from the Neurosynth database (Yarkoni et al., 2011). The ability to predict activation patterns for novel tasks based on data from other tasks was also demonstrated by Pinho et al., (Pinho et al., 2021), using the extensive Individualized Brain Charting database (Pinho et al., 2018).

In the present study, we extend these previous studies by developing cognitive encoding models using expert cognitive annotations of a broad range of tasks. We first assess the ability of individualized CEMs to predict brain activation patterns for unseen tasks, comparing generalization performance to a permutation-based null model and other benchmarks. Next, we perform hierarchical clustering on the similarity structure of CEM generalization errors to examine relations amongst ontological entities. To quantify CEM generalization across subjects, we train CEMs using the data from a single subject and then evaluate how well this model can predict the activation patterns for each of the other individuals, repeating this process for each subject. Finally, we characterize the cognitive relevance of canonical large-scale resting-state functional networks by examining the strength of learned regression weights within each network.

## 2. Methods

### 2.1. Data and code availability

The data used in this study are openly available via OpenNeuro (King et al., 2020, doi: 10.18112/openneuro.ds002105.v1.1.0). All analysis code is available at https://github.com/waltersjonathon/cognitive_encoding_models.

### 2.2. Dataset

**Fig. 1.**
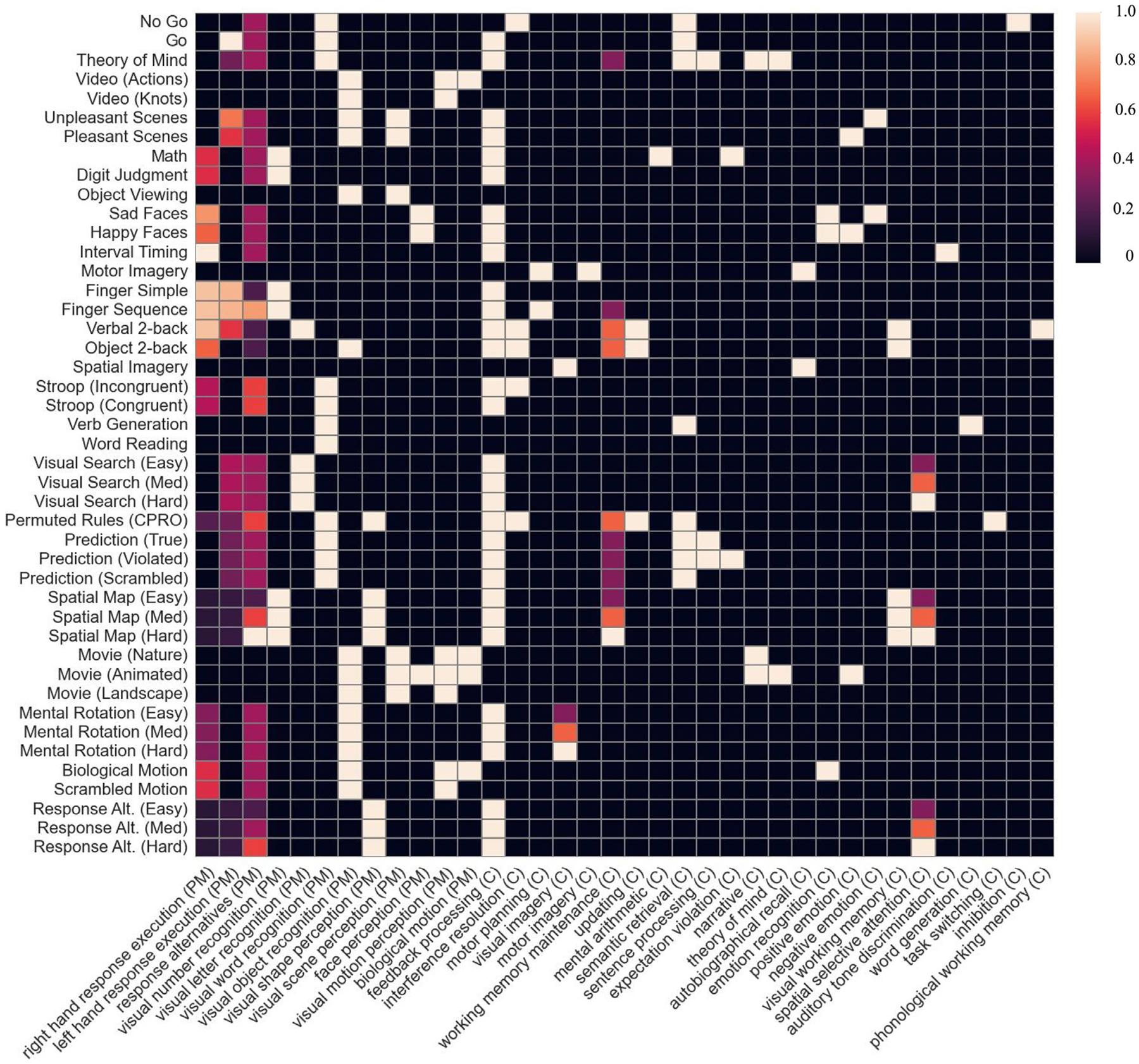
Annotation of tasks with ontological features drawn from the Cognitive Atlas ontology. “C” and “PM” indicate whether the feature was considered to be *Cognitive* or *Perceptual-Motor*.

We used the openly available Multi-Domain Task Battery fMRI dataset (King et al., 2019), designed to measure a broad range of cognitive processes. All experimental protocols were approved in the original study by the Ethics committee at Western University (Protocol #107,293), including informed consent provided to all participants. In brief, 24 healthy subjects (16 F; age: *M* = 23.8, SD = 2.6) each engaged in 47 diverse task conditions (e.g., finger tapping, movie watching, n-back, rest) across 32 ~10-minute scans. Each scan consisted of a continuous task paradigm, in which subjects performed a sequence of 17 tasks (5-*sec* instructions, 30-*sec* task execution) presented in a random order fixed across subjects (see Fig. 1 for tasks and their annotations). Subjects were scanned on two task sets (A and B), and each task set had two scanning sessions (with 8 scans per session). 16 subjects were scanned on set A in year 1 and set B in year 2, while the other 8 subjects were scanned on set A in year 2 and set B 2–3 weeks later. The two task sets partially overlapped: 29 task conditions in A and 32 task conditions in B, with 14 common to both. While the original paper considered target and non-target trials of the Verbal 2-back and Object 2-back tasks as different conditions (resulting in 4 total conditions), the current study viewed working memory load as constant across the trial types and thus considered each version of the n-back task as a single condition (resulting in 2 total conditions). Moreover, since contrasts in the present study were computed relative to the Rest condition, all analyses described herein are based on 44 unique task conditions. Finally, one subject (*sub-29)* was excluded from analyses due to failure of fMRI preprocessing.

### 2.3. fMRI data

Results included in this manuscript come from preprocessing performed using *fMRIPrep* 1.5.1rc1 (Esteban, Markiewicz, et al., 2018, 2018, RRID:SCR_016216), which is based on *Nipype* 1.3.0rc1 (Gorgolewski et al., 2011, 2018, RRID:SCR_002502). Many internal operations of *fMRIPrep* use *Nilearn* 0.5.2 (Abraham et al., 2014, RRID:SCR_001362), mostly within the functional processing workflow. The anatomical and functional data preprocessing descriptions below are adapted from automated output of *fMRIPrep*.

#### 2.3.1. Anatomical data preprocessing

For each subject, a T1-weighted (T1w) image was corrected for intensity non-uniformity (INU) with “N4BiasFieldCorrection” (Tustison et al., 2010), distributed with ANTs 2.2.0 (Avants et al., 2008, RRID:SCR_004757). The T1w-reference was then skull-stripped with a *Nipype* implementation of the “antsBrainExtraction.sh” workflow (from ANTs), using OASIS30ANTs as target template. Brain tissue segmentation of cerebrospinal fluid (CSF), white-matter (WM) and gray-matter (GM) was performed on the brain-extracted T1w using FAST (FSL 5.0.9, RRID:SCR_002823, Zhang, Brady, and Smith, 2001). A T1w-reference map was computed after registration of the T1w image (after INU-correction) using “mri_robust_template” (FreeSurfer 6.0.1, Reuter, Rosas, and Fischl, 2010). Brain surfaces were reconstructed using “recon-all” (FreeSurfer 6.0.1, RRID:SCR_001847, Dale, Fischl, and Sereno, 1999), and the brain mask was refined with a custom variation of the method to reconcile ANTs-derived and FreeSurfer-derived segmentations of the cortical gray-matter of Mind-boggle (Klein et al., 2017, RRID:SCR_002438). Volume-based spatial normalization to the *ICBM 152 Nonlinear Asymmetrical template version 2009c* (Fonov et al. 2009, RRID:SCR_008796; TemplateFlow ID: MNI152NLin2009cAsym] was performed through nonlinear registration with “antsRegistration” (ANTs 2.2.0), using brain-extracted versions of both T1w reference and the T1w template.

#### 2.3.2. Functional data preprocessing

For each of the 32 BOLD runs per subject, the following preprocessing was performed. First, a reference volume and its skull-stripped version were generated using a custom methodology of *fMRIPrep*. A deformation field to correct for susceptibility distortions was estimated based on a field map that was co-registered to the BOLD reference, using a custom workflow of *fMRIPrep* derived from D. Greve’s “epidewarp.fsl” script (https://www.nmr.mgh.harvard.edu/~greve/fbirn/b0/epidewarp.fsl) and further improvements of HCP Pipelines (Glasser et al., 2013). Based on the estimated susceptibility distortion, an unwarped BOLD reference was calculated for a more accurate co-registration with the anatomical reference.

The BOLD reference was then co-registered to the T1w reference using “bbregister” (FreeSurfer) which implements boundary-based registration (Greve and Fischl, 2009). Co-registration was configured with six degrees of freedom. Head-motion parameters with respect to the BOLD reference (transformation matrices, and six corresponding rotation and translation parameters) were estimated before any spatiotemporal filtering using “mcflirt” (FSL 5.0.9, Jenkinson et al., 2002). BOLD runs were slice-time corrected using “3dTshift” from AFNI 20,160,207 (Cox and Hyde 1997, RRID:SCR_005927). The BOLD time-series were resampled to surfaces on the following spaces: *fsaverage5*. The BOLD time-series (including slice-timing correction when applied) were resampled onto their original, native space by applying a single, composite transform to correct for head motion and susceptibility distortions. These resampled BOLD time-series will be referred to as *preprocessed BOLD in original space*, or just *preprocessed BOLD*. The BOLD time-series were resampled into standard space, generating a preprocessed BOLD run in *MNI152NLin2009cAsym* space. First, a reference volume and its skull-stripped version were generated using a custom methodology of *fMRIPrep*. Several confounding time-series were calculated based on the preprocessed BOLD: framewise displacement (FD), DVARS and three region-wise global signals. FD and DVARS were calculated for each functional run, both using their implementations in *Nipype* (following the definitions by Power et al. 2014). Three global signals were extracted from the CSF, the WM, and the whole-brain masks. Additionally, a set of physiological regressors were extracted to allow for component-based noise correction (*CompCor*, Behzadi et al., 2007).

Principal components were estimated after high-pass filtering the preprocessed BOLD time-series (using a discrete cosine filter with 128 s cut-off) for anatomical *CompCor* (aCompCor). A subcortical mask was obtained by heavily eroding the brain mask to exclude cortical GM regions. Components for aCompCor were calculated within the intersection of the aforementioned mask and the union of CSF and WM masks calculated in T1w space. Components were also calculated separately within the WM and CSF masks, and the *k* components with the largest singular values were retained, such that the retained components’ time series were sufficient to explain 50 percent of variance across the nuisance mask (CSF, WM, or combined). The remaining components were dropped from consideration.

The confound time series derived from head motion estimates and global signals were expanded with the inclusion of temporal derivatives and quadratic terms for each (Satterthwaite et al., 2013). All resamplings were performed with *a single interpolation step* by composing all the pertinent transformations (i.e. head-motion transform matrices, susceptibility distortion correction when available, and co-registrations to anatomical and output spaces). Gridded (volumetric) resamplings were performed using “antsApplyTransforms” (ANTs), configured with Lanczos interpolation to minimize the smoothing effects of other kernels (Lanczos, 1964).

#### 2.3.3. Statistical maps and parcellation

In the MDTB dataset, subjects were scanned on two task sets, separated by 2–3 weeks (*n* = 8) or a year (*n* = 16), with two scanning sessions per task set and 8 scans per session. Since the two task sets shared a subset of tasks, data for each task was available in either two or four scan sessions. Thus, for each subject, two or four statistical activation maps were estimated for each of the 44 task conditions. Maps were generated by first fitting a general linear model (GLM) to the time series of each voxel, separately for each of the 32 imaging runs. A fixed effects analysis then combined runs within sessions, and session-wise contrasts of *condition > rest* were computed for each task condition, with the resulting t-statistics converted to z-scores.

Task conditions, including the 5-second instruction screen preceding each 30-second task block, were modeled in the GLM as boxcar or event-related regressors convolved with the canonical hemodynamic response function (SPM). The duration of each regressor varied by condition (30, 16, 15, 14, 10, 5, 2, or 1 s; see Table 1 for the complete description). The other regressors included in the model were confounds estimated from fMRI preprocessing: 6 motion parameter estimates, framewise displacement, 7 cosine bases for high-pass filtering, and the first 6 components from anatomical CompCor. A first-order autoregressive model was used to model the temporal structure of the noise.

For computational speed during model training and testing, we parcellated the resulting z-maps into 1000 cortical regions, using an atlas derived from resting-state data that was optimized for both local and global spatial signal variability (Schaefer et al., 2018). In doing so, the z-score of a given region was calculated as the average across its voxels.

### 2.4. Labeling tasks with ontological feature vectors

A group of five cognitive scientists [JW, MK, PB, RI, RP] collaboratively and iteratively labeled the 44 tasks with 36 cognitive, perceptual, and motor features (Fig. 1) based on a conceptual analysis of each task. Annotations, along with entities and relations in the Cognitive Atlas, were updated throughout several iterations of the labeling process. Each feature corresponded to an entity in the Atlas, such as “working memory maintenance” (https://www.cognitiveatlas.org/concept/id/trm_55b6b9d7c9435). The resulting feature vectors captured the tasks’ partially overlapping functional requirements. Most of the features were binary (e.g., ‘Visual word recognition’, ‘Updating’, ‘Motor planning’), indicating the presence or absence of a particular operation while executing the task. Three features were parametrically coded to reflect the magnitude of their involvement: 1) ‘Response alternatives’ (0, 1, 2, 4, 6, or 7 response options), 2) ‘Left hand response execution’ (0, 1, 2, 5, 7, 8, 12, or 15 responses in a task block, and 3) ‘Right hand response execution’ (0, 1, 2, 3, 4, 5, 7, 8, 12, or 15 responses in a task block). These three features were recoded as the index of their respective rank-orderings, with 0 being the smallest value, and were then scaled to a range of 0 to 1. Additionally, all features were manually classified as being either primarily cognitive or perceptual/motor in nature (Supplementary Table 1) in order to assess the relative importance of those two classes of features in the model.

### 2.5. Cognitive encoding models (CEMs)

#### 2.5.1. Training and testing

For each subject, region-wise cognitive encoding models (CEMs) were trained to predict responses across tasks from features that captured the tasks’ psychological requirements, as formalized by an ontology. This region-wise linear mapping from ontological space to brain activation space was learned using ridge regression. In principle, once region-wise CEMs are learned, a cortical activation pattern can be predicted for any arbitrary task from its ontological feature vector.

To estimate generalization performance to unseen tasks, a leave-two-out cross-validation (CV) scheme was employed (Mitchell et al., 2008). In each CV split, region-wise CEMs were trained on 42 tasks, and the activation patterns of the two held-out tasks were predicted. Three performance metrics were then measured: two-way classification accuracy (see below), map-wise Pearson’s *r,* and *R*^*2*^.

A nested-CV scheme was necessary to select the regularization strength parameter *alpha*. The outer loop (leave-two-out CV) used a correlation-based minimum-distance classifier to classify each of the two predicted maps as one of the two true held-out maps, and an inner loop (10-fold CV) found the optimal *alpha* using Pearson’s *r* between true and predicted maps as the scoring function. The following *alpha* values were used in the parameter search: [0.001, 0.01, 0.1, 1, 2, 3, 4, 5, 6, 7, 8, 9, 10].

Map-wise Pearson’s *r* (and *R*^*2*)^ was computed for each of the two held-out tasks in each CV split (44 tasks choose 2 = 946 total splits). Since the predicted map of one task was associated with either two or four observed maps (derived from two or four fMRI imaging sessions), its map-wise correlation was taken as the average across sessions. The resulting 1892 averaged correlations across all CV splits were then averaged to obtain a single map-wise correlation for a given subject, providing a summary of estimated generalization to unseen tasks. For the noise ceiling analysis (described below) computed at the level of tasks, these 1892 values were also averaged by task.

#### 2.5.2. Two-way classification accuracy

**Fig. 2.**
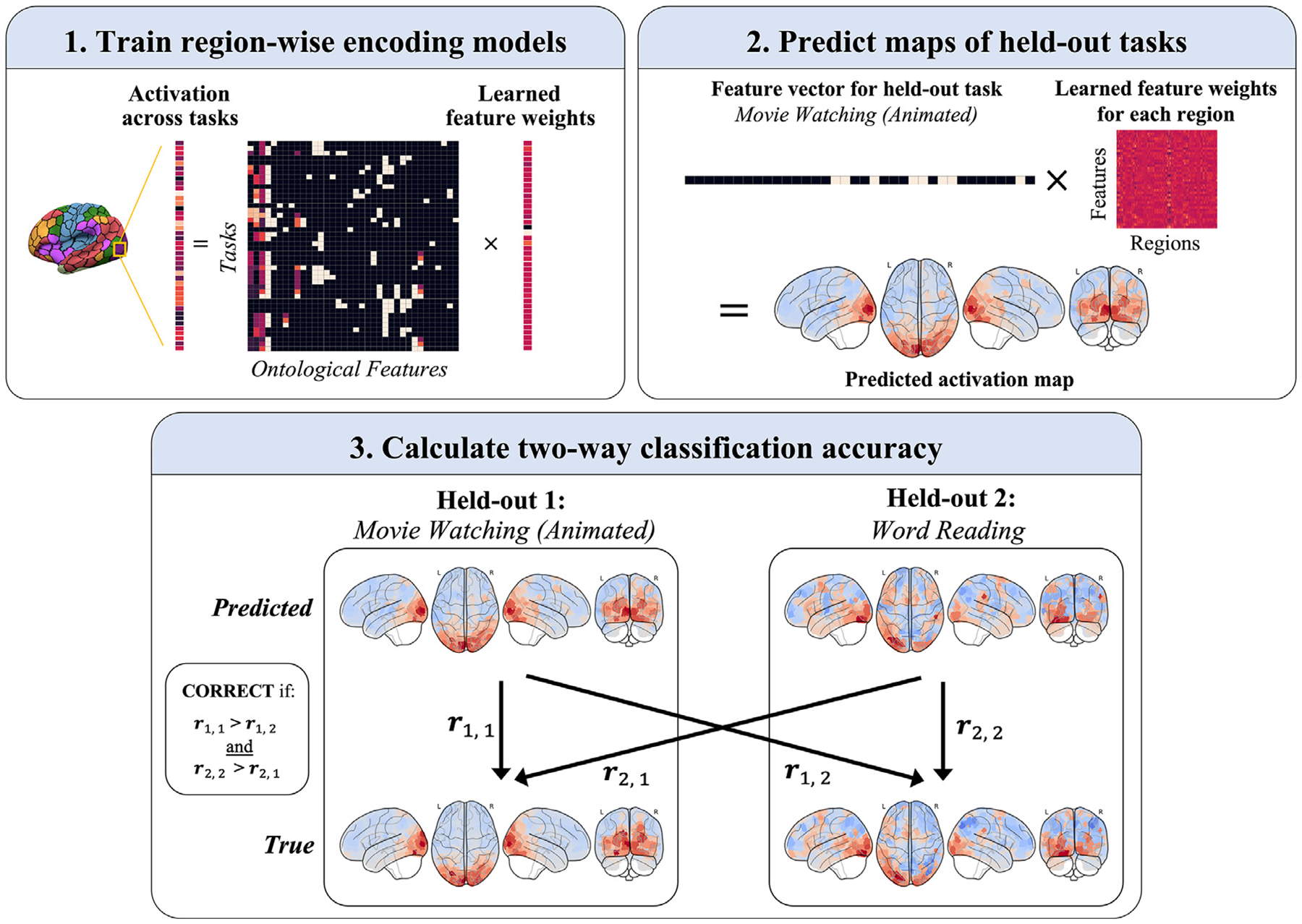
Training and testing procedure for cognitive encoding models (CEMs), showing an example cross-validation split. (1) In a leave-two-out cross-validation scheme, region-wise CEMs are trained with ridge regression to predict regional activation across 42 of 44 tasks. An inner 10-fold CV loop fits the regularization parameter alpha. An example brain region is shown. (2) Trained CEMs are then used to predict the maps of two held-out tasks. An example prediction is displayed. (3) Two-way classification accuracy for the two held-out tasks is then calculated, in which a correlation-based minimum-distance classifier assigns each predicted map to one of the held-out tasks. Accuracy is correct only if both predicted maps are correctly classified (leading to a theoretical chance accuracy of 0.25). The average accuracy across all splits is calculated per subject. Example predictions of the Word Reading (left) and Animated Movie Watching (right) tasks are shown for sub-03. Note that, for each held-out task, either two or four true maps are present (corresponding to the two or four imaging runs in which the task appeared), and the average correlation across these maps serves as input to the classifier (for brevity, these additional maps are not displayed).

Classification accuracy was computed using a leave-two-out CV scheme based on Mitchell et al. (2008). In each fold, the trained region-wise CEMs predicted the statistical activation maps of two held-out tasks. A correlation-based minimum-distance classifier, classified each of the two predicted maps as belonging to one of the two tasks. Since one predicted map was associated with either two or four observed maps (corresponding to two or four fMRI imaging sessions), the average Pearson correlation across the two or four sessions served as input to the classifier. Two-way classification accuracy was considered correct if both predicted images were correctly classified (Fig. 2), leading to a theoretical chance level of 25% accuracy.

#### 2.5.3. Noise ceiling

In general when evaluating encoding models, it is important to interpret model performance relative to the noise ceiling of the data (i.e. its reliability). While our models are trained at the level of individual brain regions, our main interest and analyses revolve around predictions for individual subjects at the level of cortical activation maps evoked by tasks. Thus, to allow for direct comparison between model performance and an upper limit of explainable variance, we quantified the noise ceiling of each task, for each subject, as the square root of the between-session reliability (Pearson’s *r*) of the activation maps (Lage-Castellanos et al., 2019). For tasks that were completed at both timepoints and thus had two between-session reliability estimates, these reliabilities were averaged in any downstream analyses.

#### 2.5.4. Task-shuffled null model

We compared CEM performance to a task-shuffled (permutation-based) null model. In each permutation, tasks were randomly remapped to one another such that each task’s feature vector was replaced by that of another task. This procedure thus targeted the relationship between tasks’ ontological features and regions’ activations. Notably, because each task had two or four corresponding rows in the original design matrix (depending on whether the task appeared in two or four imaging sessions), this method of randomizing the design matrix preserved blocks of tasks (i.e., the two or four rows of a given task all received identical feature vectors from another task); however, column densities were not preserved (i.e., feature vectors that originally appeared in two rows could appear in four rows, or vice-versa), which at least partially violated the assumption of exchangeability. Randomization and training/testing (using the same nested cross-validation procedure as the main models) was repeated for 20 iterations, and final performance metrics for each subject were calculated as the average across iterations.

### 2.6. Feature set comparison

To assess the relative importance of the Cognitive and Perceptual-Motor features in model performance, all of the main analyses presented in this paper were performed on three different feature sets: all 36 features (*All*), 24 higher-order cognitive features (*Cognitive*), and 12 perceptual-motor features (*Perceptual-Motor*). Assignment of features to these groups was based on expert judgment of authors JW and RP.

### 2.7. Hierarchical relations between ontological entities

The hierarchical organization of cognitive functions was examined based on classifier generalization errors. First, a confusion matrix was generated by calculating, for each task, how often its predicted pattern was classified correctly or was misclassified as each of the other tasks. These results were aggregated across subjects by considering all CV splits from all subjects. This confusion matrix was projected into cognitive space by multiplying the confusion matrix by the feature matrix. Next, a representational dissimilarity matrix (RDM) was constructed using 1-correlation as the distance metric. Finally, hierarchical agglomerative clustering was applied to the RDM using the UPGMA algorithm.

### 2.8. Between-subject similarity of brain function

We measured how well CEMs generalized between subjects by training CEMs on each subject individually and then testing each of these “source” subject’s CEMs on every other “target” subject. CEMs were trained on all 44 tasks and these parameters were then used to predict the activation patterns for all other subjects. Two-way classification accuracy was then calculated for every pair of tasks (analogous to the main analyses) for every target subject.

### 2.9. Using CEMs to characterize the cognitive relevance of large-scale resting state networks

Seven canonical large-scale functional resting-state networks (RSNs) (Yeo et al., 2011) were probed for their cognitive relevance using the individualized CEMs. Feature importances were estimated within each RSN by aggregating regional model coefficients across subjects from their individualized CEMs, and then aggregating across regions within each RSN. Specifically, for a given region and feature, the coefficients across subjects were first averaged. Then, for a given network and feature, these subject-averaged values were averaged across all regions within the network. This procedure allowed us to address whether spatial distributions of brain functions systematically overlapped with known functional networks.

## 3. Results

### 3.1. Cognitive encoding models generalize well to unseen tasks

#### 3.1.1. General CEM performance

**Fig. 3.**
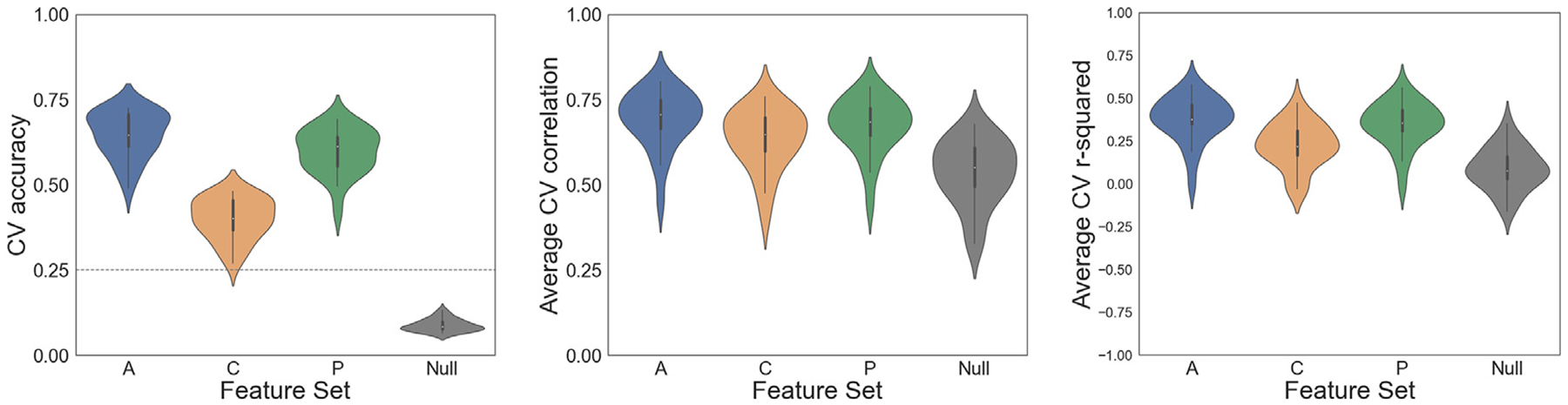
Cross-validated (CV) performance metrics for individualized cognitive encoding models (CEMs). (left) CV classification accuracy across subjects, for the full feature set (all features, ‘A’), its Cognitive (‘C’) and Perceptual-Motor (‘P’) subsets, and the permutation-based null model. Dashed line indicates theoretical chance accuracy of 25%. (middle) Average Pearson’s *r* between true and predicted statistical activation maps. (right) Average *R*^*2*^ between true and predicted statistical activation maps. To obtain the average map-wise *r* and *R*^*2*^ for a given subject, in each of the 946 CV splits *r* or *R*^*2*^ was first calculated for each of the two held-out tasks (on each of their two or four predicted maps, corresponding to the two or four imaging sessions in which the held-out task appeared), and the resulting values across all splits were then averaged.

**Fig. 4.**
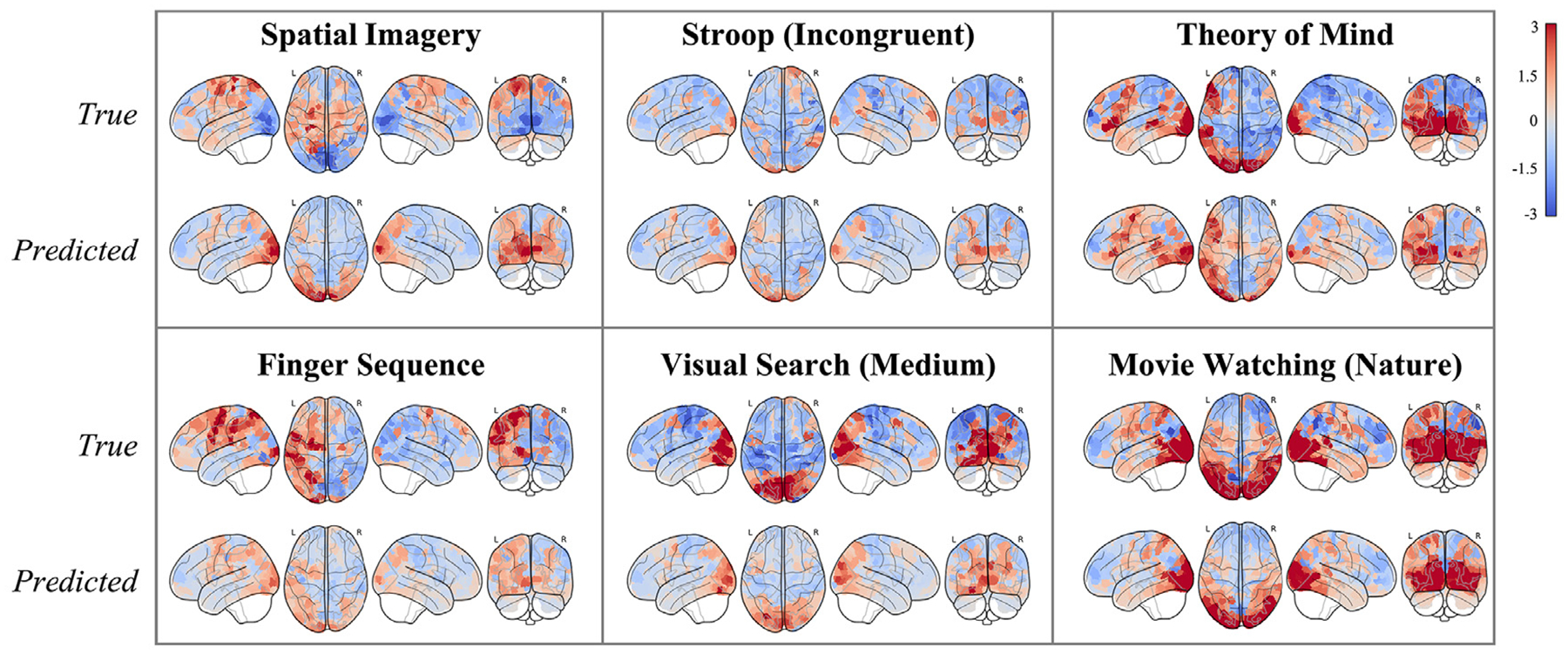
Six example out-of-sample predictions of task-evoked brain activation during cross-validation for *sub-02*. Predictions are ordered from lowest to highest correlation with the true maps: *Spatial Imagery* : −0.27, *Stroop (Incongruent)* : 0.50, *Theory of Mind* : 0.58, *Finger Sequence* : 0.61, *Visual Search (Medium)* : 0.88, *Movie (Nature)* : 0.93). Color scale indicates true and predicted z-scores, ranging from −3 to 3.

CEMs generalized well to unseen tasks (Fig. 3; example predictions in Fig. 4), with an average cross-validated two-way classification accuracy well above chance-level performance of 0.25 for all three feature sets: All (*M* = 0.65, SD = 0.07); Cognitive (*M* = 0.40, SD = 0.06); Perceptual-Motor (*M* = 0.60, SD = 0.07). The task-shuffled null model showed performance below the theoretical chance accuracy level (*M* = 0.09, SD = 0.02).

Additionally, relative to the null model, the predicted activation maps of CEMs showed stronger positive correlations with the true held-out maps: All (*M* = 0.69, SD = 0.08); Cognitive (*M* = 0.63, SD = 0.09); Perceptual-Motor (*M* = 0.67, SD = 0.08); task-shuffled (*M* = 0.54, SD = 0.09). The three feature sets also showed higher *R*^*2*^ than the null model: All (*M* = 0.38, SD = 0.13); Cognitive (*M* = 0.22, SD = 0.13); Perceptual-Motor (*M* = 0.35, SD = 0.13); task-shuffled (*M* = 0.09, SD = 0.12) (see Supplementary Fig. 1 for region-wise *R*^*2*^ values).

The null model’s classification accuracy was systematically below chance due to a bias towards predicting the mean of the training data (this also explains its relatively high *R*^*2*^ ; see Supplementary Fig. 2 for additional details).

#### 3.1.2. Feature set comparison

Next, we asked how two-way classification accuracy differed between the three feature sets. Notably, though the number of features varied across models, our cross-validation procedure provided an implicit control for this difference. A one-way repeated measures ANOVA revealed a significant difference between feature sets, F(2, 44) = 750.66, *p* < .001. A post hoc analysis with three Bonferroni-corrected paired samples t-tests revealed that All significantly outperformed both Cognitive (*t*(22) = 32.69, *p* < .001) and Perceptual-Motor (*t*(22) = 10.50, *p* < .001), and Perceptual-Motor significantly outperformed Cognitive (*t*(22) = 26.26, *p* < .001).

This pattern of results indicates that the Cognitive and Perceptual-Motor feature spaces each provided valuable information in predicting patterns of cortical activation and that CEM performance was not driven exclusively by perceptual-motor features. Thus, the inclusion of features that broadly span the space of mental functions (from lower-level perceptual-motor functions to higher-level cognitive functions) in region-wise encoding models may lead to superior performance when predicting cortical activation maps of novel tasks.

#### 3.1.3. Noise ceiling

**Fig. 5.**
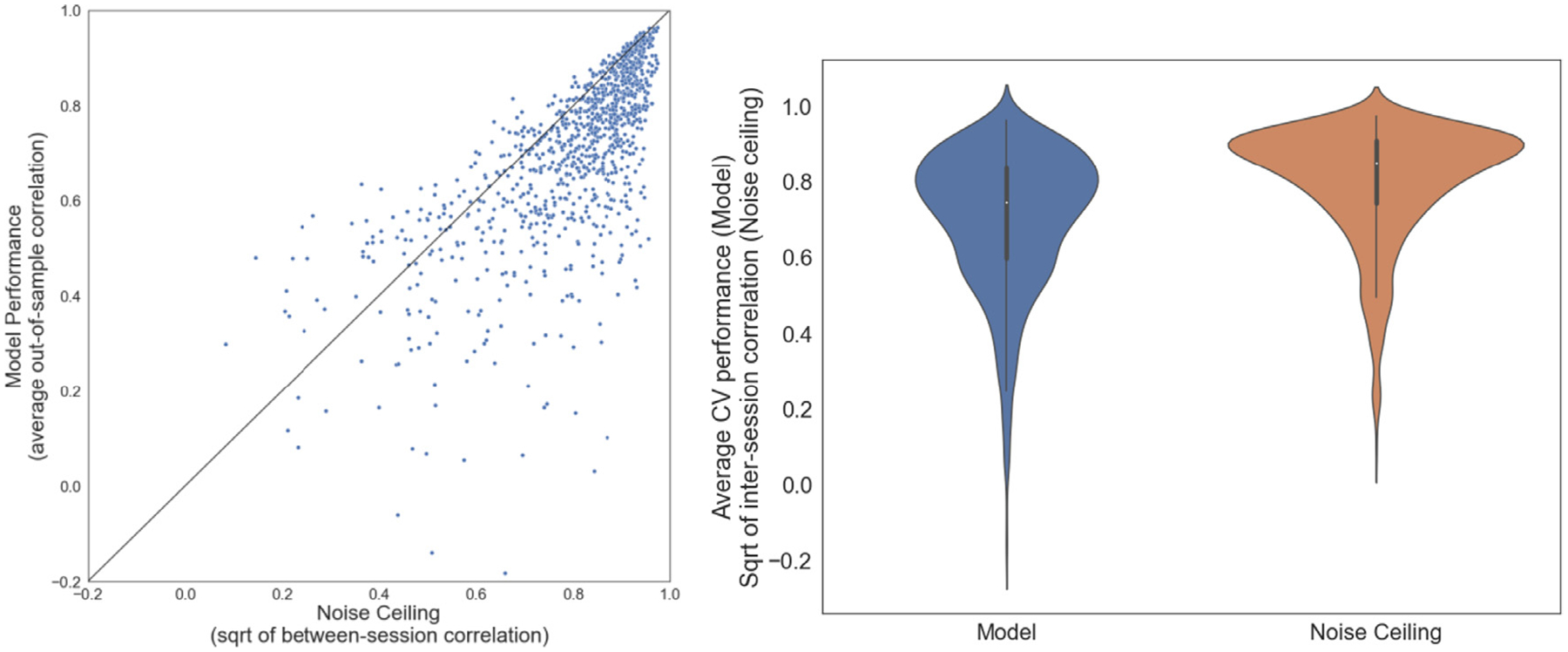
Comparison of model performance (out-of-sample correlation between true and prediction activation maps) with the noise ceiling (square root of between-session correlation). Each point represents one of 44 tasks for a given subject.

To situate the encoding model results with respect to the noise ceiling of the data, for each subject and task we estimated the noise ceiling in the activation maps by computing the square root of the test-retest reliability (Pearson’s *r*) between imaging sessions. The out-of-sample correlations for the individualized CEMs was comparable to the test-retest reliabilities, with a Pearson’s *r* of 0.70 (Fig. 5) (Model: *M* = 0.71, SD = 0.18; Noise Ceiling: *M* = 0.80, SD = 0.15). The average test-retest reliability for individual tasks ranged from 0.61 to 0.94 (Supplementary Fig. 3) and for individual subjects ranged from 0.44 to 0.90 (Supplementary Fig. 4). These results indicate that CEMs successfully learned a generalizable mapping from ontological space to brain activation space, overall approaching but generally not exceeding the noise ceiling.

The instances where the noise ceiling was exceeded (176 of 1012 task-subject pairs, or 17.4%) were driven by two related factors. First, the five subjects with the lowest average test-retest reliabilities comprised 42% of such cases, suggesting that reliability estimates may have been artifactually low in these cases. Second, there were five meta-tasks in the MDTB that each had three task conditions (i.e., Mental Rotation, Spatial Map, Visual Search, Response Alternatives, and Prediction), and these task conditions comprised 70% of such cases. Thus, for these tasks, correlations for out-of-sample predictions during cross-validation were likely boosted when one or two of the related conditions were present in the training data. However, as explained in the following section, model performance in general was not driven solely by such dependencies.

#### 3.1.4. Model performance is not driven solely by dependencies between train and test data induced by cross-validation splits

**Fig. 6.**
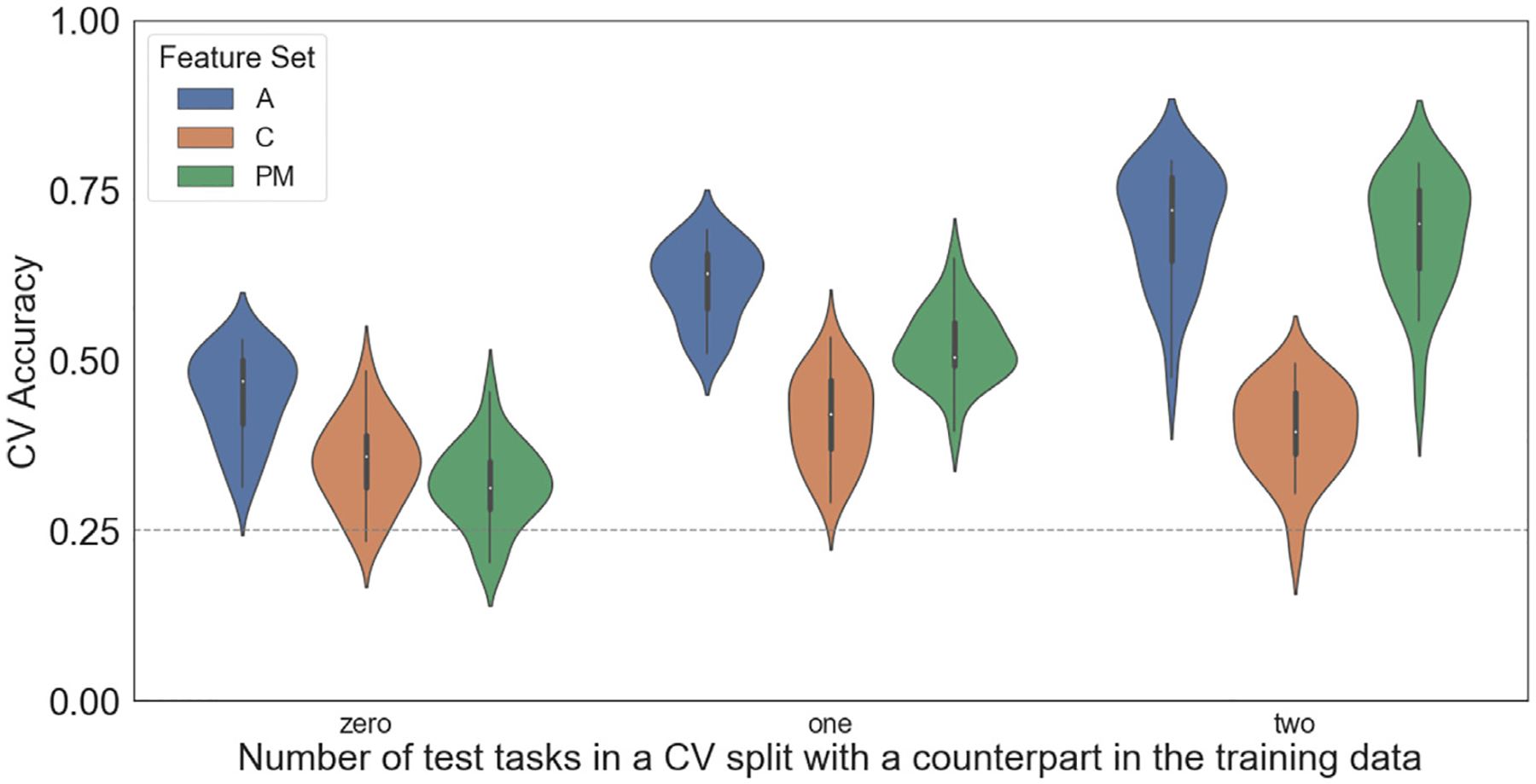
Cross-validated (CV) two-way classification accuracy for each feature set and type of cross-validation split. Some tasks defined in the encoding models are experimental conditions derived from the same meta-task (e.g., Easy, Medium, and Hard trials in the Mental Rotation meta-task), and therefore each CV split had zero, one, or two of the held-out tasks with a counterpart in the training data. Regardless of CV split type, accuracy remained above theoretical chance for all three feature sets.

As some of the tasks defined in the encoding models are experimental conditions derived from the same meta-task (e.g., Easy, Medium, and Hard trials in the Mental Rotation meta-task), we examined whether our main results were driven purely by the subset of cross-validation splits in which one or both of the held-out test tasks had counterparts in the training data (i.e., thereby increasing statistical dependence between training and testing data). We found that, for splits in which neither of the test tasks had counterparts in the training data, all three feature sets nevertheless performed above the theoretical chance accuracy level of 25% (Fig. 6), providing strong evidence that the generalizability of these encoding models was not an artifact of statistical dependencies induced by particular cross-validation splits. However, it was also clear that performance increased as the match of tasks between training and test splits increased, and that this was driven primarily by perceptual-motor features.

### 3.2. Classifier generalization errors reveal hierarchical relationships between cognitive functions

**Fig. 7.**
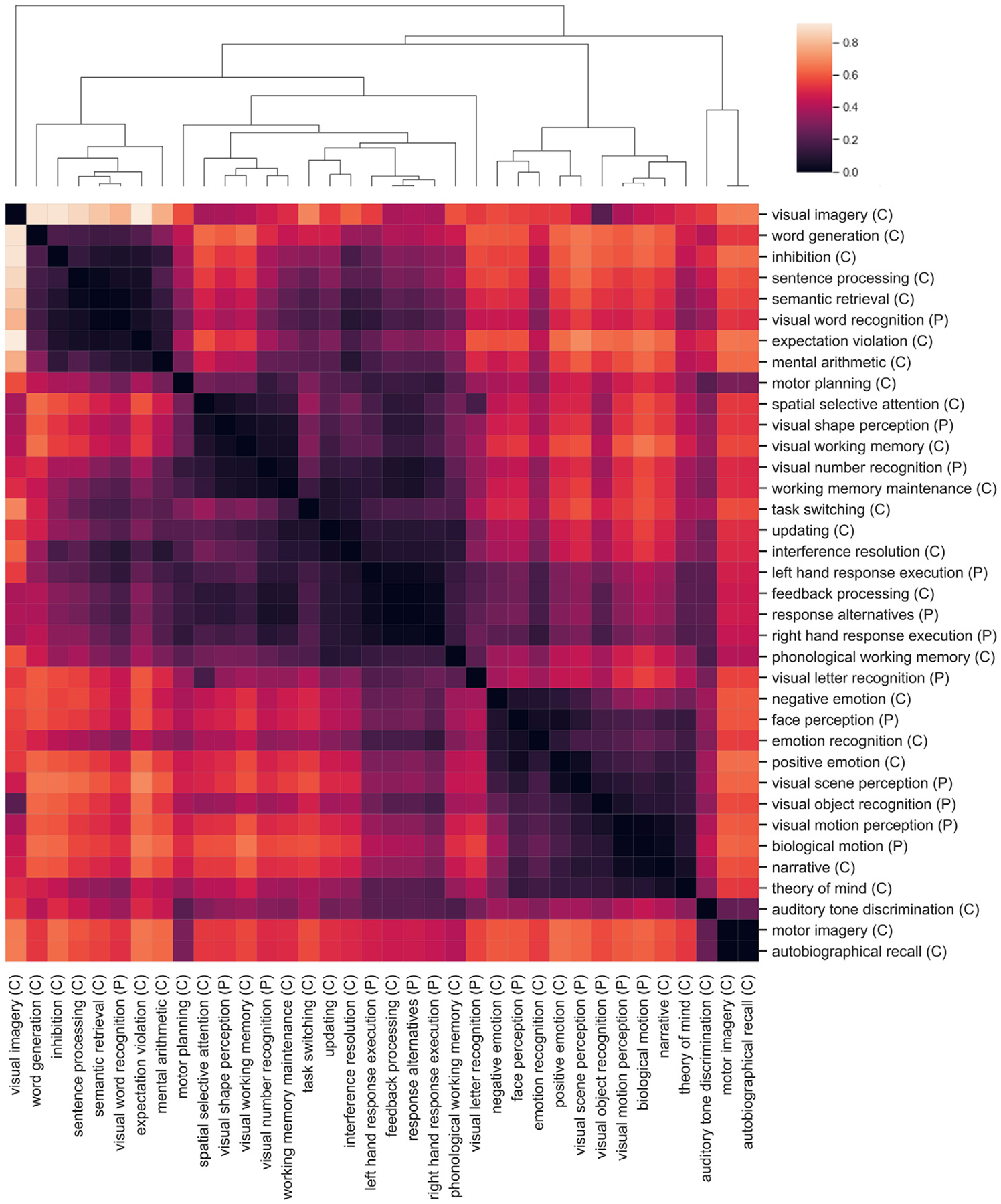
Hierarchical organization of cognitive functions based on patterns of classification errors for each task. Darker colors indicate greater feature similarity. A confusion matrix was first generated by calculating, for each task, how often its predicted pattern was classified correctly or was misclassified as each of the other tasks. Results were aggregated across subjects and CV splits. The confusion matrix was then projected into cognitive space by multiplying it with the feature matrix. Finally, a representational dissimilarity matrix (RDM) was constructed from the resulting matrix using 1-correlation as the distance metric, and hierarchical agglomerative clustering was applied to the RDM using the UPGMA algorithm.

To examine relationships between ontological features, classifier generalization errors from the individualized CEMs were aggregated by task across subjects. Fig. 7 shows a clustered representational dissimilarity matrix (RDM) for ontological entities based on classifier generalization errors.

### 3.3. Between-subject generalization

**Fig. 8.**
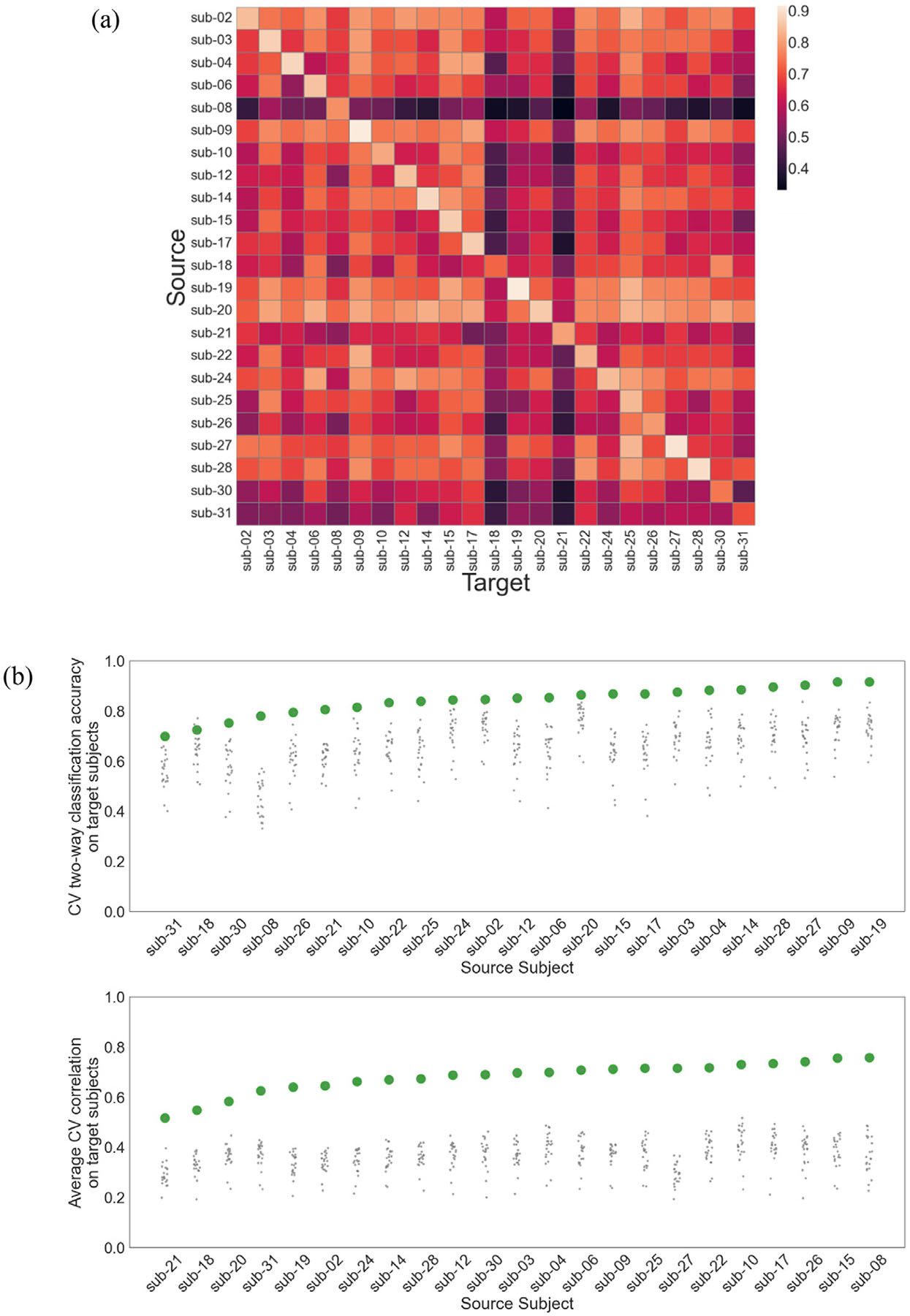
Generalization of cognitive encoding models across subjects. (a) Transfer matrix showing the degree to which individualized CEMs trained on source subjects generalize to target subjects (color bar indicates two-way classification accuracy). (b) Individual differences in two-way classification accuracy (top) and correlation (bottom), when generalizing from one source subject to all other target subjects. For each source subject, CEMs were trained on all available data, and the predictions from these models were then used in the calculation of cross-validated performance metrics for every other target subject (smaller gray dots), including the source subject (larger green dots).

We quantified how well CEMs generalized between subjects by training models on individual subjects and testing them on all others (Fig. 8 a). Subjects showed average generalization accuracies (Fig. 8 b, top) (*M* = 0.65, SD = 0.07) that were well above the theoretical chance accuracy level of 0.25 and below the self-transfer accuracies (*M* = 0.84, SD = 0.06). Average CV correlation (Fig. 8 b, bottom) on target subjects (*M* = 0.36, SD = 0.03) was lower than self-transfer correlations (*M* = 0.68, SD = 0.06).

### 3.4. Mapping spatial distributions of CEM coefficients onto large-scale resting state networks

After validating the individualized CEMs, we sought to use the trained models to characterize the function of large-scale resting-state networks (RSNs) as an additional way of validating the results with regard to known functional associations. To do so, regional model coefficients from subjects’ CEMs were aggregated across subjects and then within RSNs, allowing for estimates of feature importance within each RSN.

**Fig. 9.**
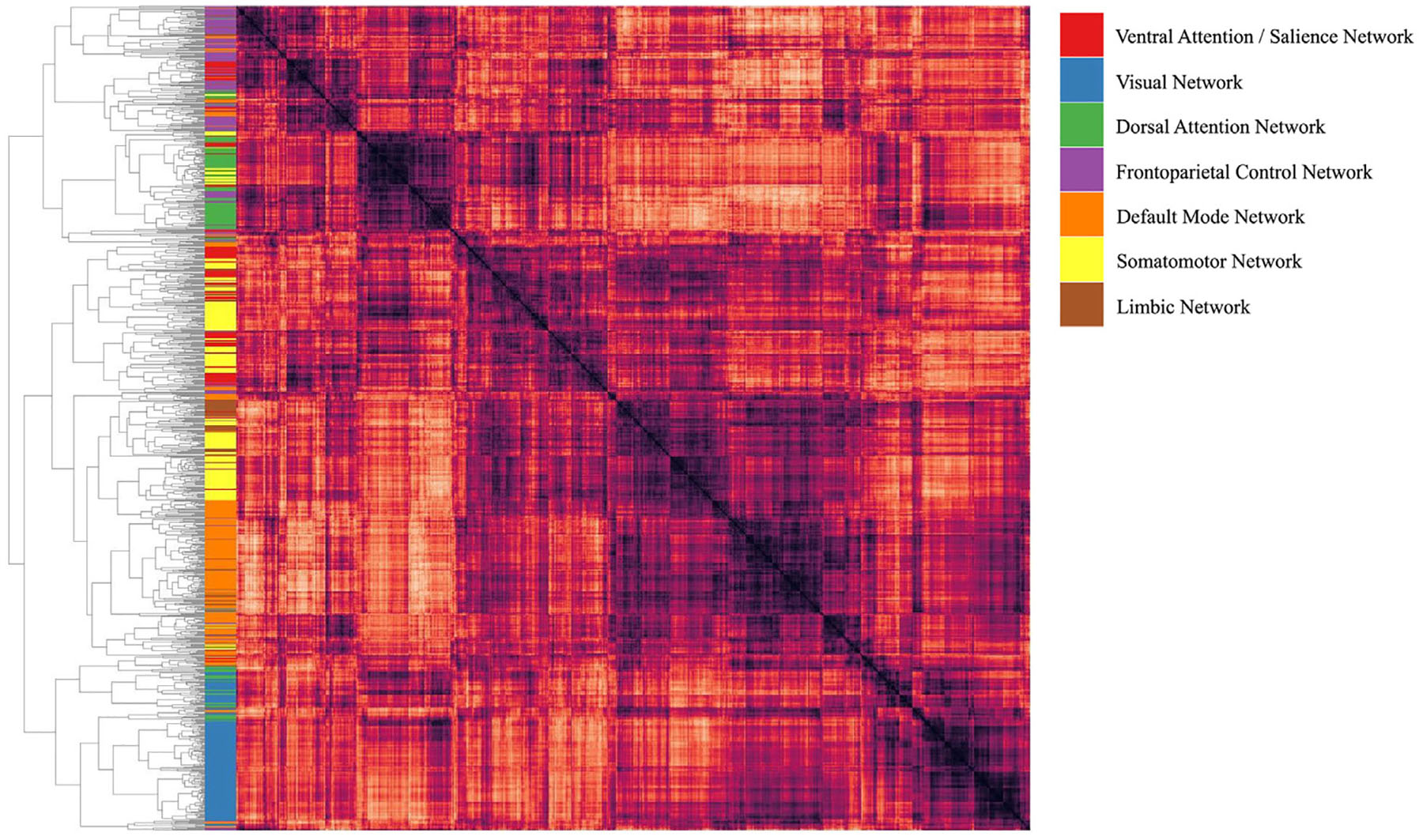
Clustering (UPGMA method) of 1000 brain regions based on the similarity (Pearson’s *r*) of their model coefficients with other regions. These clusters exhibit a tight correspondence with known large-scale functional resting-state networks (indicated by color).

**Fig. 10.**
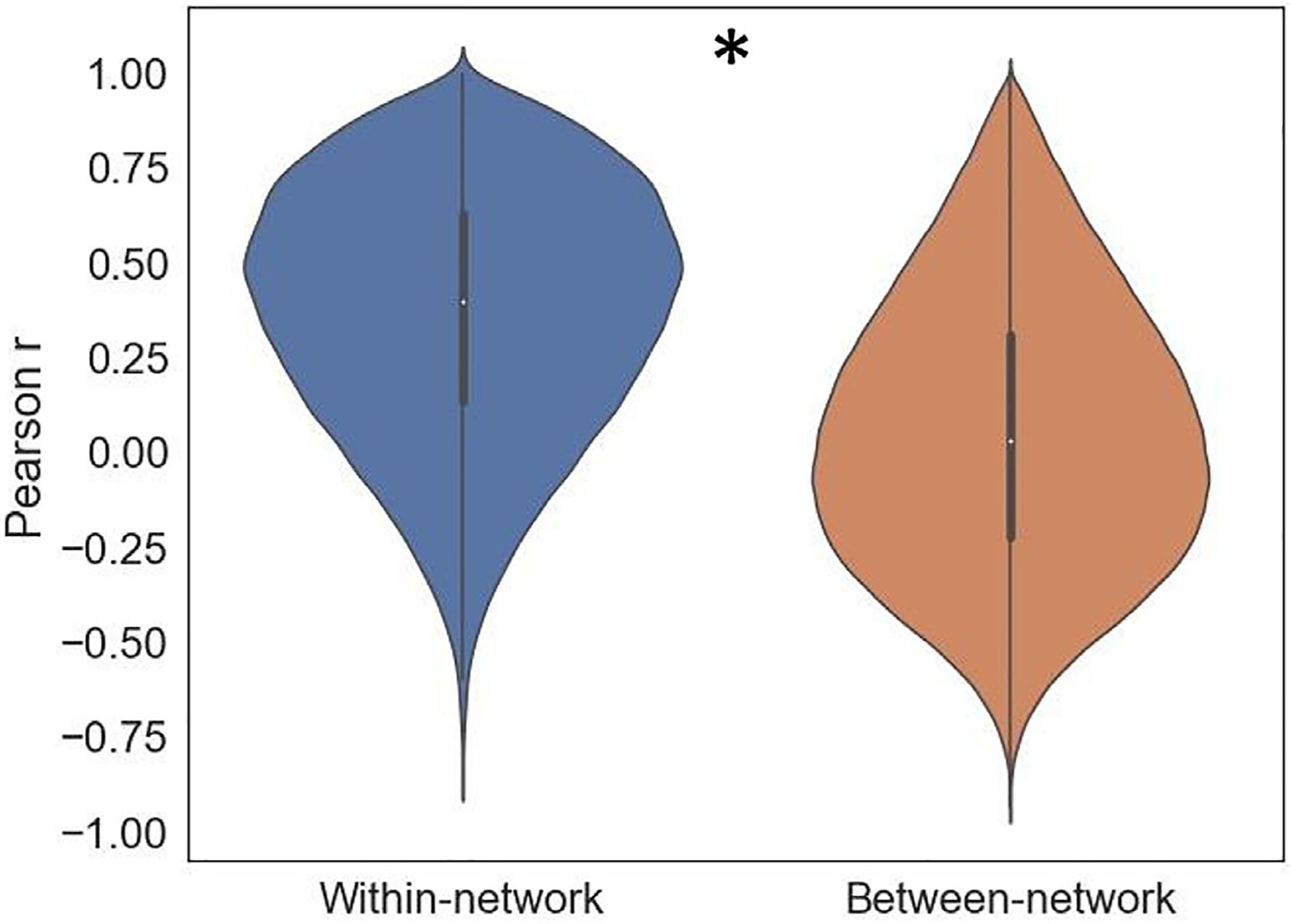
Similarity of CEM coefficients for pairs of regions residing in the same or different large-scale resting-state network. Pairs of regions belonging to the same resting-state network showed more positively correlated feature weight vectors than pairs of regions from different networks. Asterisk indicates permutation-based statistical significance (100 iterations, *p* < .01).

First, we tested the hypothesis that RSNs possess a degree of functional specialization, finding that CEM coefficients were more similar between regions in the same network than in different networks (Figs. 9 and 10) (within-network: *M* = 0.37, *SD* = 0.33; between-network: *M* = 0.05, *SD* = 0.36; *p* < .01, based on permutation tests randomizing *within* and *between* labels for 100 iterations). While this result is expected given that our encoding models as well as the definitions of regions and networks we used are both activity-based, it supports the hypothesis of functional specialization in large-scale RSNs while also grounding their function in an interpretable and data-driven manner.

**Fig. 11.**
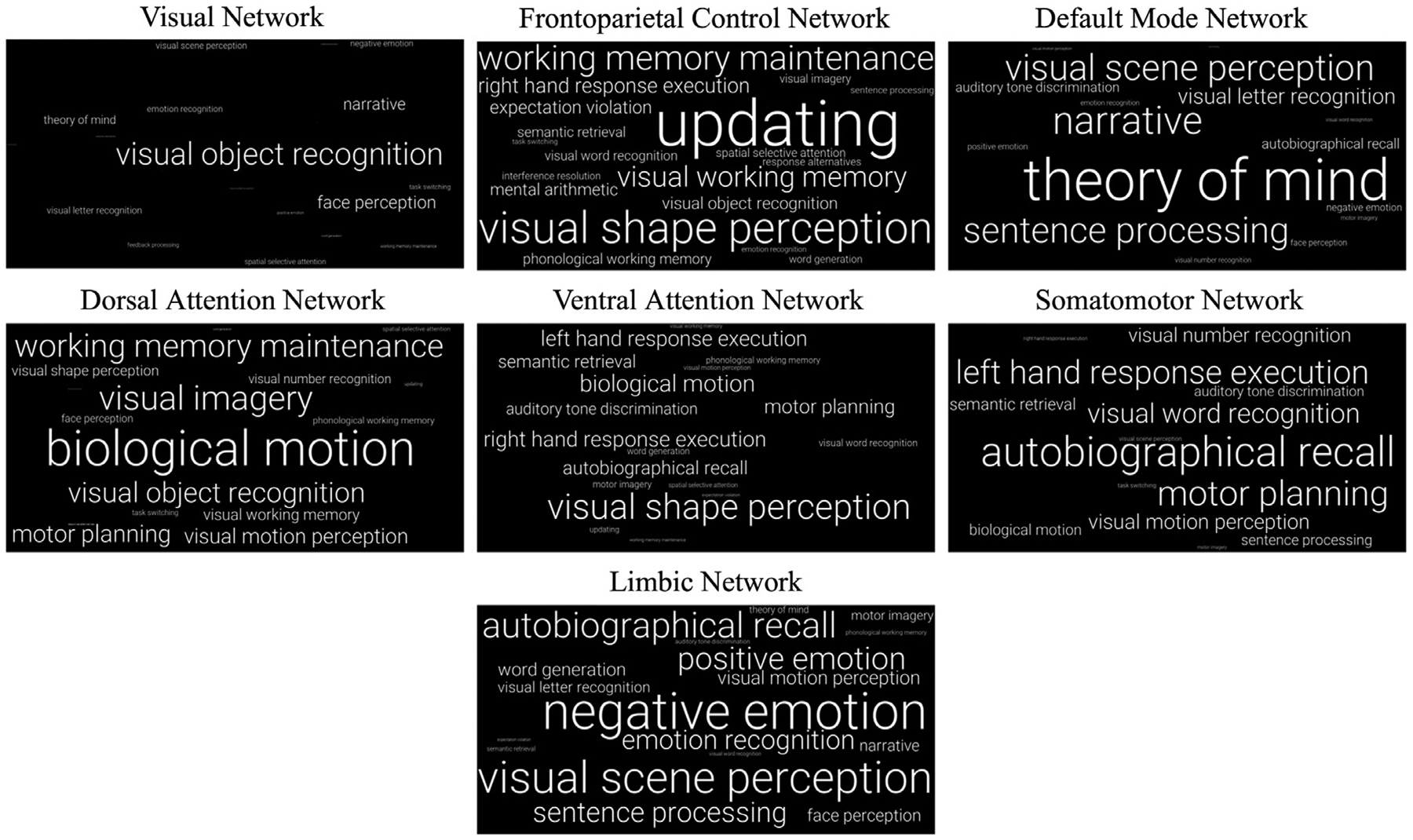
Word cloud representations of relative feature importances in seven canonical large-scale resting-state networks. For each feature, encoding model coefficients were first averaged across subjects by region, and then averaged across regions within each network. Average coefficient magnitudes are mapped directly to font size; a feature with twice the magnitude of another feature is twice the font size. Only features with positive weights are included.

Next, we visualized the function of each network by generating word cloud representations of relative feature importances (Fig. 11). Many of the dominant features across the networks reflect known functional associations. For example, the most positively weighted feature in the Visual Network (VN) is *visual object recognition* and in the Default Mode Network (DMN) is *theory of mind*. Interestingly, a few of the dominant features across networks did not match expectations based on prior work (e.g., *autobiographical recall* in the Somatomotor Network).

## 4. Discussion

In the present work we developed encoding models based on a cognitive ontology and applied them to an fMRI dataset that included a broad range of cognitive task conditions, with the goal of predicting activation patterns for held-out task conditions. The results demonstrated that these models can effectively predict activation patterns for novel tasks based on the annotation of cognitive processes engaged by the task, both within individuals and across individuals. The amount of variance accounted for across tasks by the predictive model varied, in some cases accounting for nearly all of the explainable variance in the maps. Assessment of the model parameters in relation to well-established large-scale brain systems demonstrated a mapping of functions largely consistent with known functional neuroanatomy.

The present findings extend previous work that had established the ability of encoding models to predict task activation maps for broad sets of cognitive tasks (Pinho et al., 2021; Nakai and Nishimoto, 2020), by demonstrating the ability to use expert annotations of cognitive functions as the basis for the encoding model. This provides the potential to use cognitive encoding models to test cognitive theories; whereas cognitive theories rarely make specific predictions regarding locations of brain activation, they nearly always make predictions regarding the specific processes engaged by a particular set of tasks, and hence the similarity or overlap of task-related activation maps. By implementing encoding models based on competing cognitive theories and testing their predictive accuracy on out-of-sample data, this approach has the potential to adjudicate theoretical questions using neuroimaging data, thus addressing the longstanding question of whether neuroscience data can inform cognitive theories (e.g., Coltheart 2006).

### 4.1. Future directions

The present approach could be used to learn a data-driven cognitive ontology that optimally predicts activation on new tasks. Although the generation of theories with scopes that are broad enough to span all of cognition is a major challenge, this approach can still be applied to more restricted functional domains with the goal of increasing scope over time. Accordingly, future studies using this approach could potentially leverage unsupervised learning applied to the task behavior (Eisenberg et al., 2019), additional information regarding the brain networks engaged by those tasks (Nakai and Nishimoto, 2020), or information from computational models fit to those tasks (Stocco et al., 2021 ; Anderson et al., 2008). Alternatively, completely unsupervised methods could be used to learn a novel “cognitive basis set” optimized solely for prediction of activation maps. Another possibility is to use structural equation modeling on the covariance matrix between cortical activation maps to construct, test, and refine different latent representations of the underlying data-generation process.

While the use of data-driven approaches to refine existing ontologies is appealing, it is challenging because there are multiple factors that must be considered, which include: the ontology itself, the mapping of the ontology to tasks, the ontological breadth of the measured tasks, the learning algorithm, and the quality of the neuroimaging data. For example, if it is observed that Theory of Mind (ToM) is a poor predictor, it could be the case that a) ToM is an ill-defined construct, b) ToM is not mapped appropriately to tasks, c) there is an insufficient breadth of tasks that engage both ToM and mixtures of other constructs (such that the effect of ToM cannot be disentangled from that of other constructs), d) the chosen learning algorithm fails to capture the true underlying relationship between ToM and cortical activation, and/or e) the quality of the data acquired on the ToM tasks is poor.

The CEMs presented in this study model cortical activation univariately at the level of brain regions, whose collective predictions as an ensemble are then used for task classification. To more directly model patterns of brain activation, future work should also consider multivariate approaches that predict whole-brain responses from cognitive features, such as partial least squares or redundancy analysis. Another possibility is to use a CEM to inform how much difference one would expect between cortical activation patterns, as measured by an appropriate distance metric (e.g., a visual and an auditory task should be more different than two visual tasks). This type of prediction can be accomplished with multivariate distance matrix regression (Zapala et al., 2006), which has already seen application to fMRI data (Shehzad et al., 2014).

Relatedly, while explaining differences in statistical activation maps is a valuable first step in building CEMs, future work should consider bypassing the use of general linear model estimates of task activation altogether, opting instead to more directly relate ontologies to minimally preprocessed BOLD time courses. This strategy of directly mapping ontological entities to brain dynamics may ultimately provide the most illuminating application of CEMs, in part due to the reduction of bias that may arise if a modeler (however implicitly) uses their own ontological knowledge in constructing the task regressors.

Finally, the present study assessed the ability of a formal ontology to predict cortical activation patterns for unseen tasks, while holding the learning algorithm constant. Although this is a necessary first step in a proof-of-concept study for the utility of ontology-based cognitive encoding models, future work should systematically examine how variability in algorithmic decisions (e.g., using different linear or nonlinear modeling frameworks) contributes to the predictive capacities of CEMs.

### 4.2. Limitations

While the present work showed that a feature space based on expert manual annotations of cognitive functions affords high predictive accuracy for unseen tasks, the labor-intensive nature of the labeling method limits its scalability. The labeling method can be thought of as a function from feature space to tasks, and, in order to generalize to new tasks, the same function needs to be applied to any new tasks. Thus, to increase consistency and scalability, future work will need to explore automated labeling schemes that operate upon feature spaces of cognitive processes. Unfortunately, standard text-mining approaches are likely too imprecise to provide the level of functional detail needed to develop such models, but one promising approach is to harness methods for automatic knowledge extraction or for human-in-the-loop programmatic or semi-supervised labeling (e.g., Ratner et al., 2017). Such approaches will likely be required to generalize from the current limited annotations available to the broad range of tasks needed to develop “cognition-wide” models.

Another limitation of the current work involves simplifying assumptions regarding how the features relate to tasks. It was assumed that all subjects performed tasks using the same mixture of psychological functions (i.e., what differed across subjects was the region-wise encoding of these features). Instead, it is likely that the psychological functions actually used to perform a given task differs across subjects. Future work could explore ways of optimizing the personalization of such labels, such as using models of task performance to infer task strategies (e.g. Roy et al., 2021). Relatedly, this work is limited in its modeling of regional activation in terms of linear combinations of the psychological features. Future work should consider exploring interactions between features, for example by explicitly including interaction terms in a linear model or by using models capable of capturing non-linear interactions. Despite these simplifying assumptions, CEMs were still able to learn a generalizable mapping from ontological features to brain activation quite well, approaching the noise ceiling.

Finally, because the parcellation scheme used in this study computed regional activity by averaging across voxels, we caution readers that our analyses provide little, if any, interpretive value at the level of individual voxels.

## 5. Conclusion

Using a uniquely rich fMRI dataset, in which individuals were densely sampled performing 44 diverse tasks, we demonstrated the predictive and interpretive utility of ontology-based feature spaces in encoding models that generate subject-specific cortical activation patterns for unseen tasks. These results build on previous work that uses datasets of diverse tasks for functional brain mapping, providing further evidence for the utility of cognitive ontologies in consistently mapping cognitive functions to tasks and in selectively associating cognitive functions with their neural bases.

## Supplementary Material

1

## Figures and Tables

**Table 1:** Task conditions, associated meta-tasks, and task regressor durations used in first-level modeling of fMRI data.

Task condition	Meta-task	Duration
Rest	Rest	30
Spatial Imagery	Spatial Imagery	30
Motor Imagery	Motor Imagery	30
Verbal 2-back	Verbal 2-back	30
Object 2-back	Object 2-back	30
Object Viewing	Object Viewing	30
Theory of Mind	Theory of Mind	30
Interval Timing	Interval Timing	30
Movie (Nature)	Nature Movie	30
Movie (Landscape)	Landscape Movie	30
Movie (Animated)	Animated Movie	30
Permuted Rules (CPRO)	CPRO	30
Word Reading	Words	16
Verb Generation	Words	14
Digit Judgment	Numbers	15
Math	Numbers	15
Finger Sequence	Finger Tapping	15
Finger Simple	Finger Tapping	15
Video (Actions)	Video Action-Knots	15
Video (Knots)	Video Action-Knots	15
Scrambled Motion	Motion Perception	15
Biological Motion	Motion Perception	15
Visual Search (Easy)	Visual Search	10
Visual Search (Med)	Visual Search	10
Visual Search (Hard)	Visual Search	10
Mental Rotation (Easy)	Mental Rotation	10
Mental Rotation (Med)	Mental Rotation	10
Mental Rotation (Hard)	Mental Rotation	10
Response Alt. (Easy)	Response Alternatives	10
Response Alt. (Med)	Response Alternatives	10
Response Alt. (Hard)	Response Alternatives	10
Spatial Map (Easy)	Spatial Map	10
Spatial Map (Med)	Spatial Map	10
Spatial Map (Hard)	Spatial Map	10
Prediction (Scrambled)	Prediction	5
Prediction (Violated)	Prediction	5
Prediction (True)	Prediction	5
Instructions	Instructions	5
Stroop (Congruent)	Stroop	2
Stroop (Incongruent)	Stroop	2
Pleasant Scenes	Scene Viewing	2
Unpleasant Scenes	Scene Viewing	2
Happy Faces	Face Viewing	2
Sad Faces	Face Viewing	2
Go	Go No-Go	1
No Go	Go No-Go	1

## Data Availability

Please see Data/Code Availability Statement. Multi-Domain Task Battery (MDTB) (Reference data) (OpenNeuro)

## Data and code availability

The data used in this study are openly available via OpenNeuro (doi: 10.18112/openneuro.ds002105.v1.1.0). All analysis code is available at https://github.com/waltersjonathon/cognitive_encoding_models.
