# Supplementary material for: Predicting brain activation maps for arbitrary tasks with cognitive encoding models
Source: Neuroimage. Author manuscript; Available in PMC 2023 May 7. (PMC9981816; doi:10.1016/j.neuroimage.2022.119610)
Supplement: 1 [file NIHMS1848618-supplement-1.docx]

**Appendix. Supplementary materials**

| **Cognitive** | **Perceptual-Motor** |
| --- | --- |
| feedback processing | left hand response execution |
| interference resolution | right hand response execution |
| motor planning | response alternatives |
| visual imagery | visual word recognition |
| motor imagery | visual letter recognition |
| working memory maintenance | visual number recognition |
| updating | visual object recognition |
| mental arithmetic | visual scene perception |
| semantic retrieval | visual motion perception |
| sentence processing | biological motion |
| expectation violation | visual shape perception |
| narrative | face perception |
| theory of mind |  |
| autobiographical recall |  |
| emotion recognition |  |
| positive emotion |  |
| negative emotion |  |
| visual working memory |  |
| spatial selective attention |  |
| auditory tone discrimination |  |
| word generation |  |
| task switching |  |
| inhibition |  |
| phonological working memory |  |

**Supplementary Table 1.** Classification of ontological features as either primarily Cognitive or Perceptual-Motor in nature.


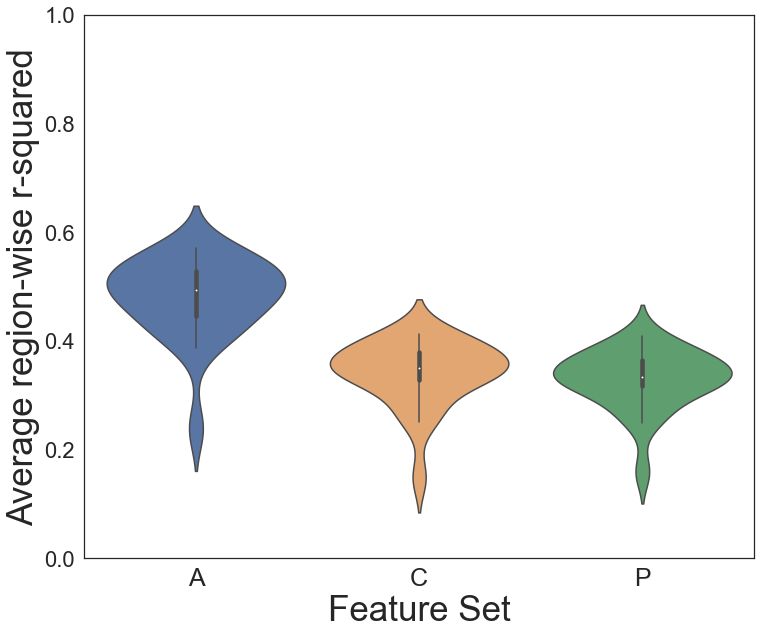

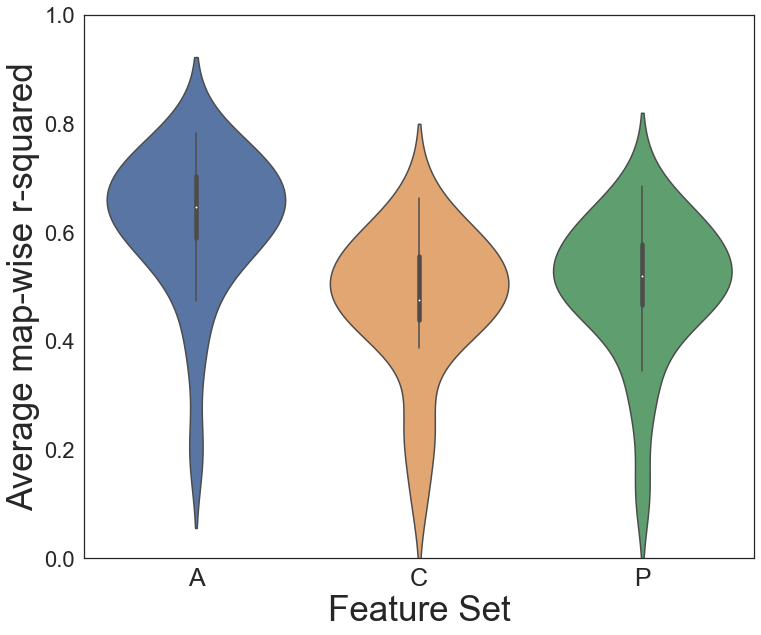


**Supplementary Figure 1.** Distributions of subjects’ average region-wise *R^2^* (i.e., between true and predicted statistical activations for each region) and average map-wise *R^2^* (i.e., between true and predicted statistical activation maps) (*right*), for the full feature set (all features, ‘A’), and its Cognitive (‘C’) and Perceptual-Motor (‘P’) subsets. These metrics were calculated for CEMs trained on all available data. Region-wise *R^2^*: All (M = 0.48, SD = 0.07), Cognitive (M = 0.34, SD = 0.06), Perceptual-Motor (M = 0.33, SD = 0.05). Map-wise *R^2^*: All (M = 0.62, SD = 0.13), Cognitive (M = 0.47, SD = 0.13), Perceptual-Motor (M = 0.50, SD = 0.13).


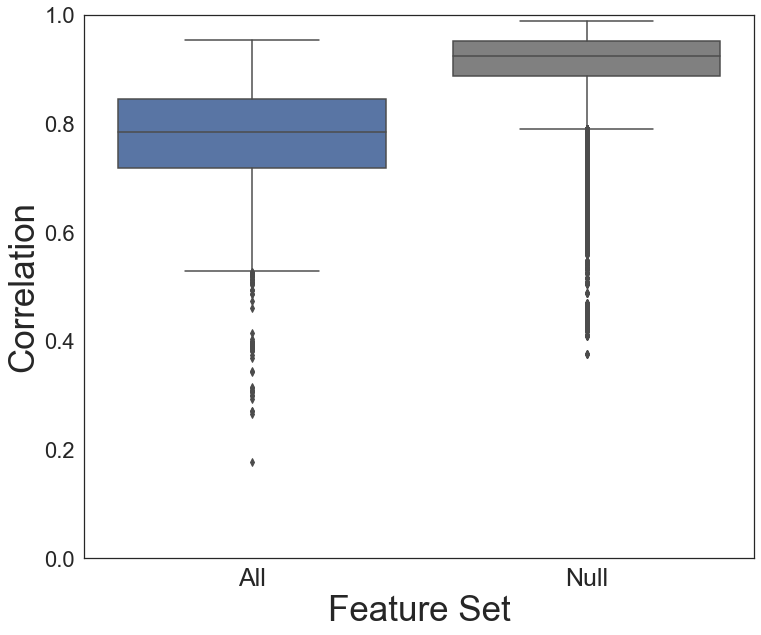

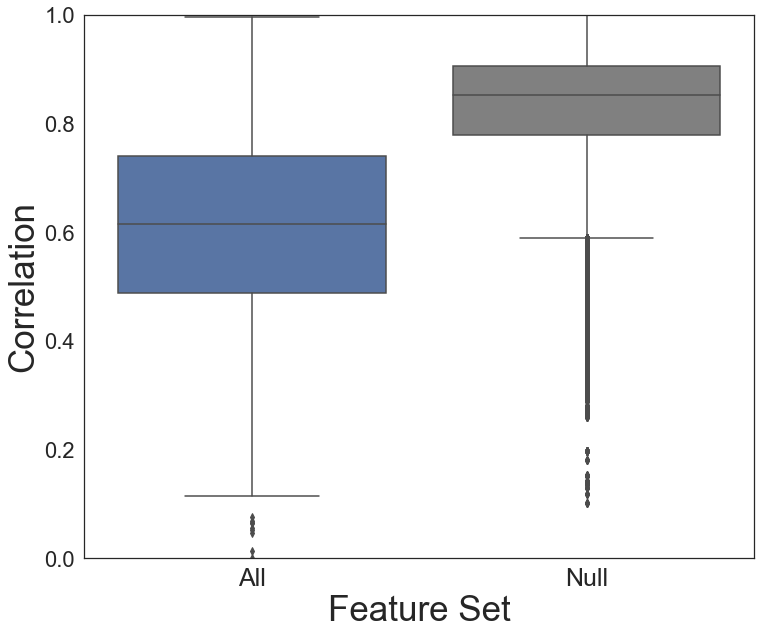


**Supplementary Figure 2.** In the main manuscript, the correlations of the null model’s predictions with the held-out data may appear to be higher than expected. However, this result is explained by regularization biasing the null model toward predicting the mean of the training data. To demonstrate this for an example subject (*sub-02*), we performed an analysis identical to that of Section 3.1.1 in the main paper and examined all cross-validation splits for 20 iterations of the task-shuffled null model (946 CV splits per iteration) and, for comparison, the *All* feature set. The null model’s predictions during cross-validation were highly similar to the mean of the training data (All: M = 0.76, SD = 0.11; Null: M = 0.91, SD = 0.06) (*left*), and the null model’s two predictions in a given cross-validation split were also highly similar to one another (All: M = 0.60, SD = 0.19; Null: M = 0.83, SD = 0.11) (*right*). This result additionally explains why the null model systematically performs at a below-chance cross-validated accuracy of 0.09 (where chance is 0.25) – at the limit, if the two predicted images are exactly equal, then accuracy in our two-way classification scheme would be 0% (since classification is correct if and only if the correlation of *each* map with its true map is greater than its correlation with the other held-out map).


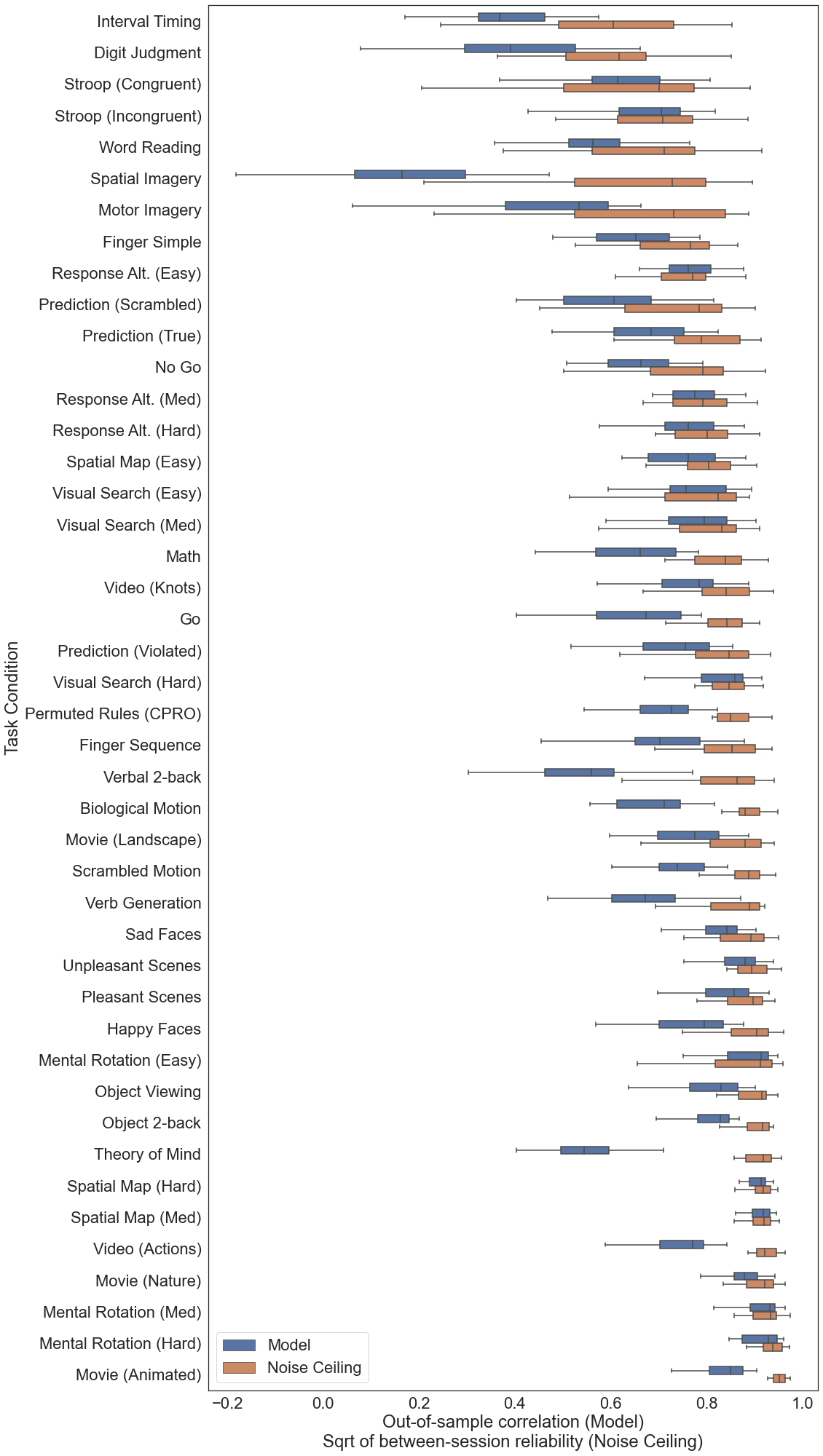


**Supplementary Figure 3.** Comparison of model performance to the noise ceiling, aggregated by task.


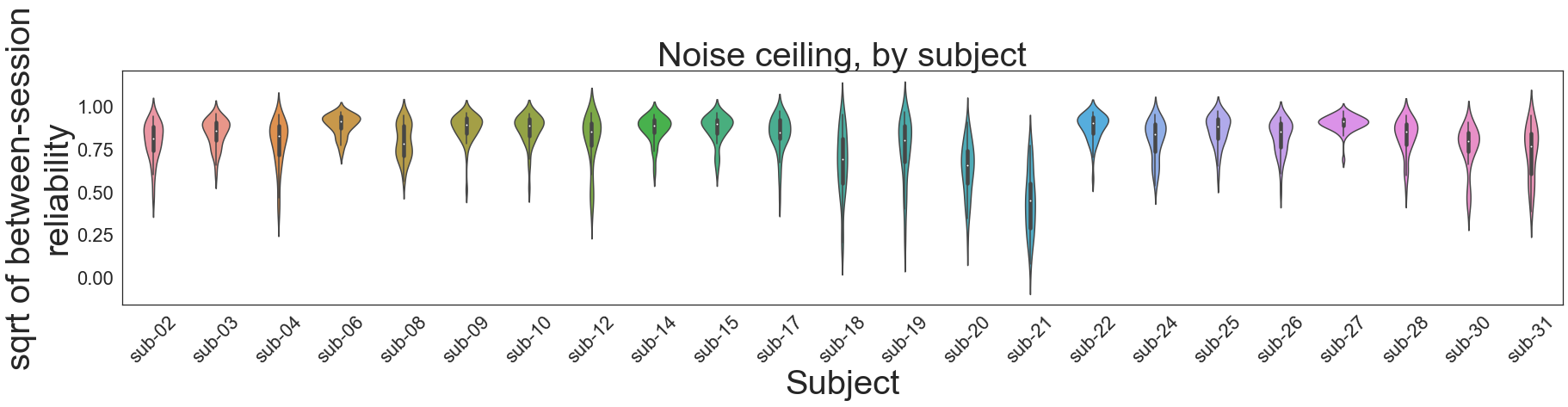


**Supplementary Figure 4.** Noise ceiling distributions (over tasks), for each subject.
